# The PyRosetta Toolkit: A Graphical User Interface for the Rosetta Software Suite

**DOI:** 10.1371/journal.pone.0066856

**Published:** 2013-07-09

**Authors:** Jared Adolf-Bryfogle, Roland L. Dunbrack Jr.

**Affiliations:** 1 Institute for Cancer Research, Fox Chase Cancer Center, Philadelphia, Pennsylvania, United States of America; 2 Drexel University College of Medicine, Program in Molecular and Cell Biology and Genetics, Philadelphia, Pennsylvania, United States of America; Universität Erlangen-Nürnberg, Germany

## Abstract

The Rosetta Molecular Modeling suite is a command-line-only collection of applications that enable high-resolution modeling and design of proteins and other molecules. Although extremely useful, Rosetta can be difficult to learn for scientists with little computational or programming experience. To that end, we have created a Graphical User Interface (GUI) for Rosetta, called the *PyRosetta Toolkit*, for creating and running protocols in Rosetta for common molecular modeling and protein design tasks and for analyzing the results of Rosetta calculations. The program is highly extensible so that developers can add new protocols and analysis tools to the PyRosetta Toolkit GUI.

## Introduction

The Rosetta Molecular Modeling suite is a collection of command-line-only applications encompassing approximately 1.7 million lines of C++ code. Within the suite there are numerous applications for modeling and design ranging from minimization of the scoring function to enzyme redesign. Rosetta is an extremely useful piece of software for a variety of molecular modeling tasks [1], [2], [3], [4], [5], [6].

Due to the wealth of useful C++ classes within Rosetta and the ease of Python programming for beginners and advanced users alike, Sergey Lyskov and the PyRosetta team created Python bindings for Rosetta [7]. PyRosetta allows direct access to nearly all Rosetta functions and classes using Python scripts, programs, and the interactive IPython/Python terminal.

To allow beginning users, including molecular biologists with little or no computational experience, to use Rosetta with ease, we have created a Graphical User Interface (GUI), which we call the *PyRosetta Toolkit*, using PyRosetta as the underlying Rosetta code. The PyRosetta Toolkit was created with code simplicity in mind, allowing users to add their own functions, menus, and windows. Although stand-alone Foldit [8], [9] can be used to run some basic Rosetta algorithms such as repacking and minimization on a single model, many modeling tasks are either difficult or non-existent in FoldIt. As such, we believe the PyRosetta Toolkit represents the first major GUI to the full functionality of the Rosetta software suite.

A complete overview and description of the PyRosetta Toolkit GUI code base and a tutorial on how to add to the GUI can be found online through the PyRosetta website (www.pyrosetta.org/documentation) and in the GUI's documentation directory. Here we provide an introduction to the GUI and an overall description.

## Results

**Figure 1:**
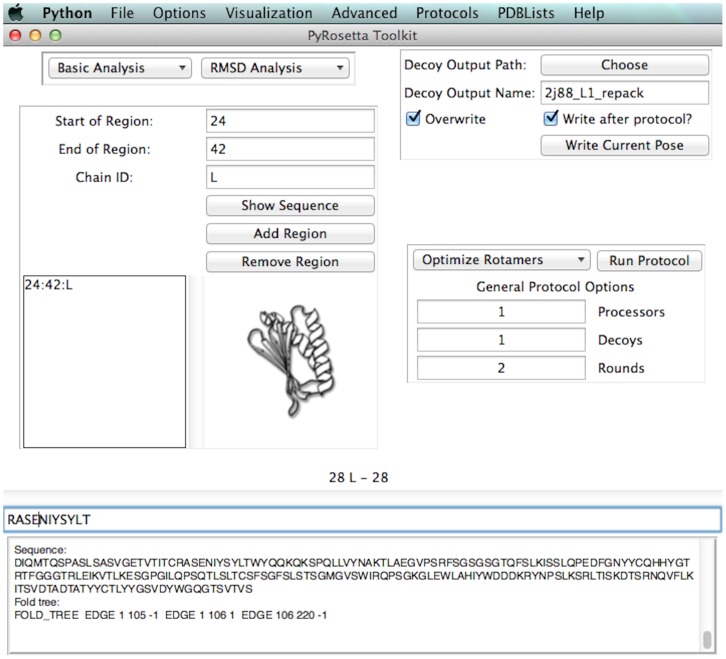
PyRosetta Toolkit GUI. *Top-Left*: Energy and RMSD analysis functions. *Bottom-Left*: Region selection. *Top-Right*: Output options. *Bottom-Right*: Decoy and Protocol options as well as some common modeling protocols.

The PyRosetta Toolkit is composed of two main areas – the *main window* and the *main menu*. The main window, shown in Figure 1, allows users to specify protein regions and output options, perform quick analyses, or run standard protocols such as relaxing or repacking structures and regions. The main menu houses many additional functions and more advanced Rosetta methods and protocols. In addition, PyMOL [10] visualization of poses, coordinate changes, and energetics through the PyMOLMover [11] are integrated into the GUI.

The main window (Figure 1) acts as a place for the user to prepare or quickly analyze a protein of interest as well as to setup decoy and output options for the session. Main functions in this window encompass region selection where a user can: choose multiple loops, chains, and termini; control decoy output, energy, and RMSD analysis; and perform common protocols such as energy minimization or rotamer repacking through both Rosetta and SCWRL4 [12]. In addition, it has a textbox where most Rosetta and Python output has been redirected to make it easier to observe results of an analysis, protocol, or file load. The sequence of the selected region is also given, and clicking next to a residue will show the PDB number and chain as well as the internal Rosetta residue numbers, which are used in a number of Rosetta applications outside of the GUI.

The menus in the PyRosetta Toolkit are as follows.

### *File Menu:*

The File Menu allows the user to load a structure from a PDB file or directly from the Protein Data Bank, prepare a PDB for use in Rosetta, save and load GUI sessions, and import or export a variety of Rosetta filetypes (Table 1).

**Table 1:** Function Overview.

**Input filetypes**	
PDB File	Rosetta Loop File
RCSB PDB ID	ParameterPath list
PDBList	GUI Session
**Output filetypes**	
PDB File	
FASTA for protein and/or regions	Rosetta Flag File
Rosetta Loop File	SCWRL Seq File
Rosetta Resfile	Parameter PathList
Rosetta Blueprint file	GUI Session
**Protocols**	
Fixed Backbone Design	Rotamer Repacking (With SCWRL Integration)
Low Resolution Docking	Low Resolution Loop Modeling (CCD + KIC)
High Resolution Docking	High Resolution Loop Modeling (CCD + KIC)
FastRelax	Grafting
ClassicRelax	FloppyTail
Energy Minimization	
**Server Links**	
ROSIE [32]	Robetta – Fragments [35]
Rosetta Backrub server [36]	Robetta – Interface Alanine Scan [37]
Rosetta Scaffold Select [38]	Robetta – DNA Interface Scan
**Analysis**	
RMSD Tools	Packing Analyzer
Energy Analysis	Design Result Breakdown
Interface Analyzer	Rotamer/Per-Residue analysis
Loop Analyzer	Arbitrary data insertion into B-Factor column
VIP Analyzer	
**PDBList Functions**	
Design Result Breakdowns	Top % or # by energy
Rescore PDBList	Energy vs RMSD
FASTA output of proteins and regions	
**PDB Prep functions**	
Water removal	Insertion code expansion
HETATM removal	Residue and atom renaming
Renumber from 1	Detection of unknown residues/Loading of params for off-by-default residues
**Other**	
Constraints	Scorefunction Creation
Non-Canonical AA integration	Resfile Creation
Variant + NCAA Mutagenesis	

**Figure 2:**
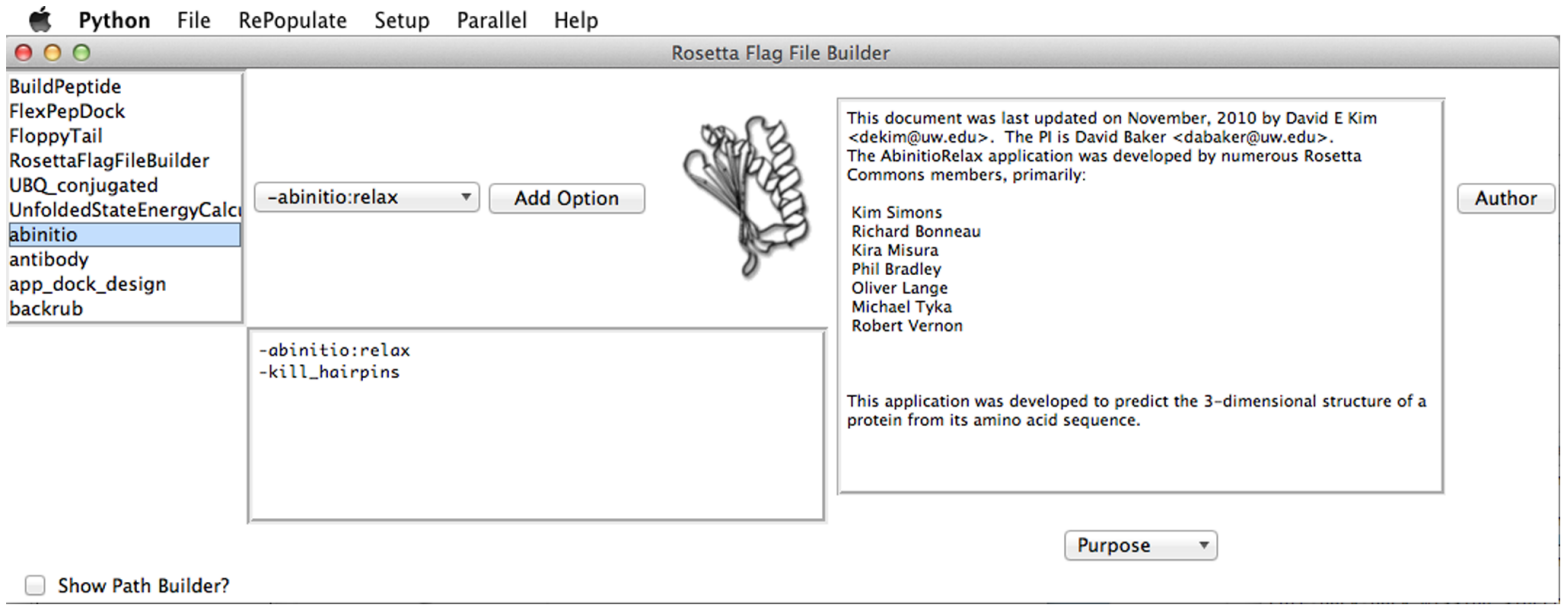
Rosetta Flag File Builder. *Top-Left*: All Rosetta applications found in a user-specified directory. *Top-Middle*: All options parsed from Rosetta Doxygen documentation housed in rosetta_source. Clicking an option gives a description, if any is found, in the textbox on the right. *Bottom-Middle*: Text window that functions in building a config file. Adding an option will add it to this textbox, while the PathBuilder allows users to search for various files and add them to the textbox. *Right*: Information textbox which gives information on individual options as well as each major component of Rosetta Doxygen documentation (Purpose, Unparsed Options, Code and Demo, References, Algorithm, Limitations, Modes, Input Files, Tips, Expected Outputs, and Post Processing).

In addition, the RosettaFlagFileBuilder GUI can be launched using this menu. This GUI is independent of PyRosetta and functions in helping a user create a Rosetta command line flag file, run Rosetta applications, and launch them on a cluster using qsub, a commonly used job submission and queuing system for cluster computers (Figure 2). It parses Rosetta Doxygen documentation for recommended options/values and the many other options available for each C++ application. A user can explore the documentation for the many applications within Rosetta interactively, as well as get information and default values for each option of each application. Functions are available for constructing or loading a flag file (usable by Rosetta command-line applications), running them locally, or submitting them to the qsub queue.

### *Options Menu:*

The Options Menu allows the user to set the number of processors to use, setup the main score function, and interact with the Rosetta options system.

**Figure 3:**
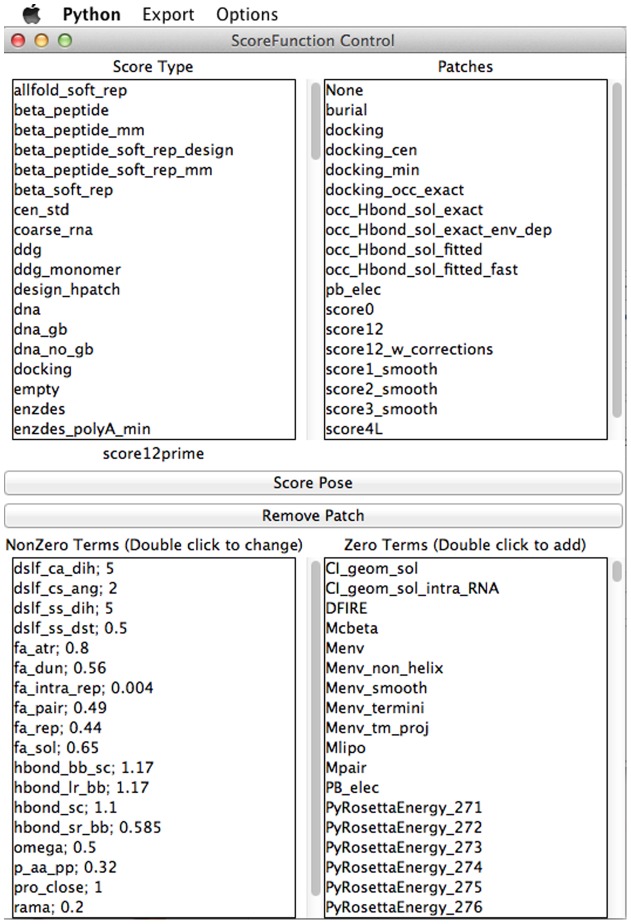
Score Function Control and Creation. *Top:* All score function weights and patch files found in the Rosetta chemical database. These weights and patches can be set as the current GUI score function or saved as the default. *Bottom-Left:* Terms in the current score function and their associated weights. Weights can be changed by double-click. *Bottom-Right:* All Rosetta energy terms can be enabled at a certain weight by double-click.

The score function window (Figure 3) allows a user to choose any weight set or patch in Rosetta, as well as modify the weight of any score term or add any score term that is not being used to the current score function. This score function is then used by any function in the GUI that requires one and can be modified at any time. A user can also save the new score function or set the GUI to use a particular set of weights by default.

General Rosetta options, such as -dun10 (the 2010 Dunbrack rotamer library [13]) or -ex2 (extra chi2 rotamers), can be set through a window where a user can select from a few common options, enter custom ones, save and load a set of options, or set defaults for the GUI.

### *Visualization Menu:*

The Visualization Menu allows the user to integrate modeling tasks directly with PyMOL using Rosetta's PyMOLMover. A user can set Rosetta to continually send models to the PyMOL program upon structural change or send the current structure (or pose in Rosetta parlance). The small Advanced PyMOL Visualization window allows users to color and label per-residue energies based on the score function or individual score term and send other useful information including DSSP secondary structure, identified hydrogen bonds, and the polar identity of each residue. This window becomes an integral part of interactive Rosetta modeling through the PyRosetta Toolkit. Note that PyMOL needs to be run separately alongside the GUI to take advantage of these visualization tools.

### *Advanced Menu:*

**Figure 4:**
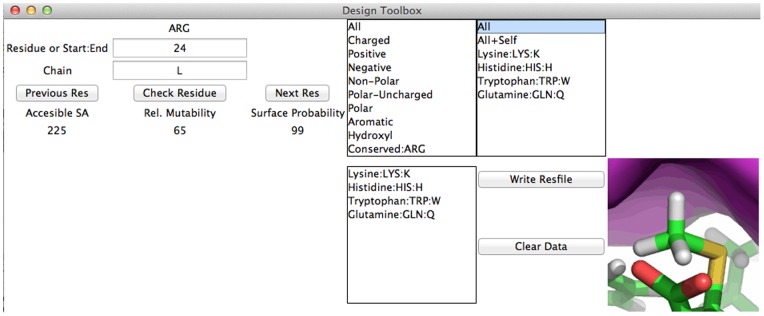
Design – Setup Resfile. *Left*: A resfile for design is constructed for individual residues or stretches of residues. Biochemical data is given for each current residue. *Right*: Selections of residue types. Once selected, individual residues in the category are added to the box on the right. Added residues are found in the lower box, where the current designable residues can be edited or cleared.

The Advanced Menu houses a variety of sub-windows and useful functions for analyzing Rosetta results. Four Rosetta-specific analyzers are implemented, including the Void Identification and Packing Analyzer (VIP) [14], Packstat [15], InterfaceAnalyzer [6], and LoopAnalyzer [6]. A user can also enable constraints for the pose and score function in the menu. A sub-window for setting up and exporting a Rosetta resfile, a file for specifying mutatable residues for a design run, is shown in Figure 4. Commonly used data for accessible surface area, surface probability [16], and relative mutability [17] are given for each current residue and potential mutant. In addition, a user can select all conservative mutations for a given position or a range of positions as well as all residues of a specific type (hydrophobic, hydrophilic, charged, etc.). A Rosetta resfile for the pose can then be exported, and the fixed-backbone protocol can be run from within the GUI, making setting up and running the Rosetta design protocol easier.

Two other integral sub-windows are housed in the Advanced Menu. These include the Ligand/Post-Translational Modification (PTM)/Non-canonical Amino Acid (NCAA) Manager and a window for per-residue control and analysis.

**Figure 5:**
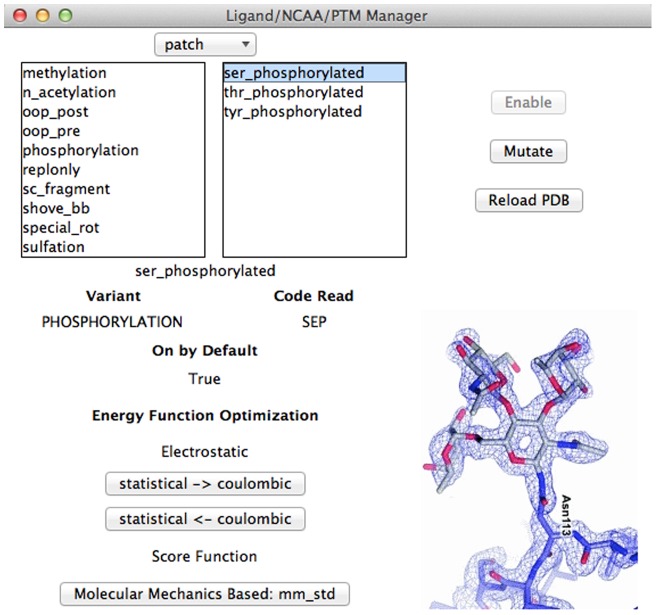
Ligand/NCAA/PTM Manager. *Top*: Selection is grouped first by patch/ligand/polymer, then by specific biochemical property. *Middle*: Rosetta related information is given about the particular selection. *Bottom:* Functions for optimizing the current energy function for use with ligands, non-canonical amino acids (NCAA), and post-translational modifications (PTM).

The Ligand/PTM/NCAA Manager (Figure 5) deals with the various parameter and patch files housed in the Rosetta chemical database. Due to memory restrictions, not all of these are enabled by default in Rosetta. Besides functioning to enable these non-canonical amino acids, post-translational modifications, and ligands that the parameter files describe, a user can explore these files in a way that enables identification of the three letter residue code Rosetta reads, determination of whether it is on by default, and of what variant type it is if any. In addition, functions that change the score function to model the physical properties of the non-standard residues or residue modifications, such as the mm_std score function developed by Renfrew et al.[18], are provided. Users may also mutate any residue to these non-canonical amino acids from this window, as long as they are enabled and the appropriate rotamer library has been included.

**Figure 6:**
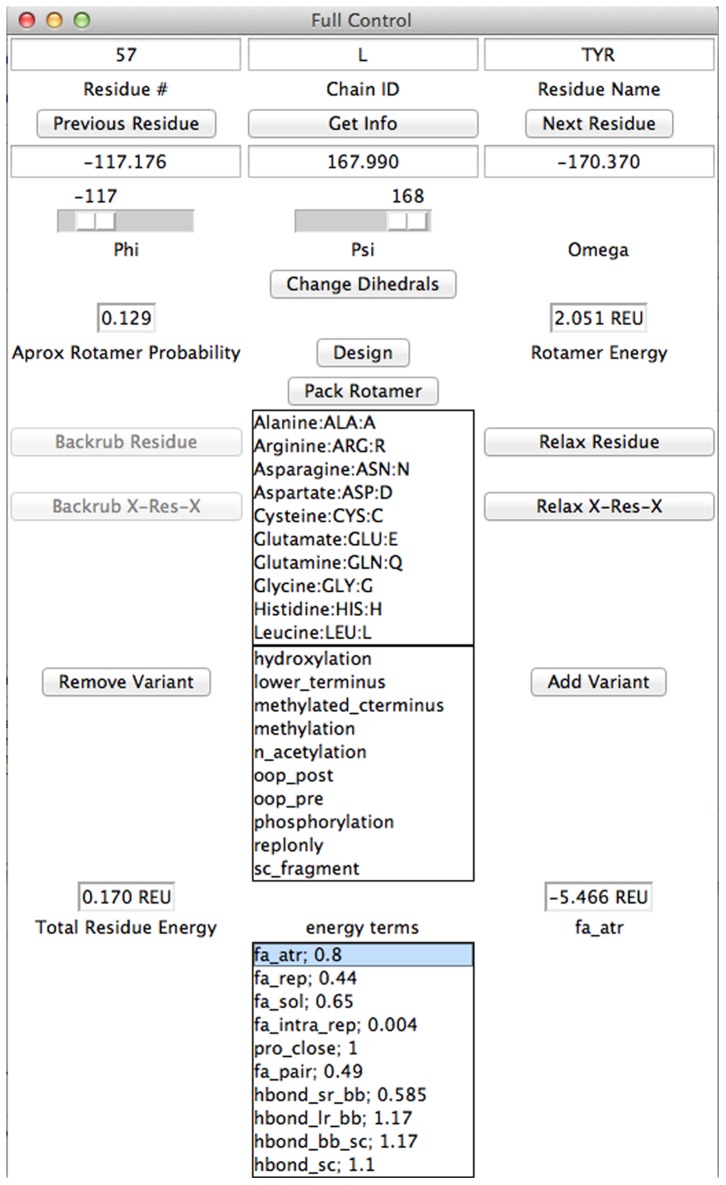
Per-Residue Control and Analysis. A collection of functions for analyzing, modeling, and designing individual residues. Per-residue energies, probabilities, and dihedral angles are given. Variants may be added or removed from residues, any residue may be designed or mutated, and individual rotamers may be optimized.

Finally, the Per-Residue Control and Analysis Window (Figure 6) allows a user to manipulate, design, and analyze individual residues of a pose. Per-residue information includes rotamer energy, approximate rotamer probability calculated by *p* = e^−*E*^, residue energy, and energy of the residue by individual score term. Users can change individual dihedral angles, mutate to any canonical amino acid, add variant types such as phosphorylations or acetylations, and repack rotamers and relax individual residues and residues in the vicinity of the chain.

### *Protocols Menu:*

Protocols in the GUI are handled by Python's multiprocessing module, allowing the user to specify the number of processors for production runs. Many protocols have their own setup windows associated with them. Currently available protocols include fixed-backbone design [2], [19], [20], [21], FastRelax [22], ClassicRelax [23], rotamer repacking [21], [24], Floppytail [25], grafting, high- and low-resolution docking [26], [27], [28], [29], and high- and low-resolution loop modeling with both CCD [9], [28], [30] and KIC [31]; with more to be added in the future. In addition, the protocols menu has links to Rosetta online servers such as ROSIE [32] and Robetta [33].

### *PDBLists Menu:*

A PDBList is a very simple text file listing a path to a PDB file on each line. This PDBList is generally used for analysis of the large number of decoy files created in a typical Rosetta simulation. Methods for loading, creating, and using this list to analyze and group results are housed in the PDBLists Menu.

Through a function in the menu, a user can create a PDBList from all the coordinate files that match user-defined filename identifiers in a given directory or recursively. This PDBList can then be used not only for the GUI but also for command-line Rosetta runs using the in: file:l flag designation. A PDBList becomes a major point of analysis after a Rosetta protocol run when a user is left with thousands upon thousands of decoy structures, even those in multiple directories with identical PDB filenames.

Using the PDBList, a user can output a FASTA-format file containing the sequence for each PDB specified or each region specified, which can then be used by many online servers for further analysis. If a user has results from a design simulation, a design breakdown can be performed that analyzes percentages of each residue type of each region specified and outputs data into a text file, an SQLITE3 database format, and auto-generated graphs in PDF format from the R program [34]. This can be extremely useful for rational design. For energy analysis, a PDBList can be rescored or a Rosetta scorefile can be read. A user can then get the top score or best-scoring poses by number or percent. These energies are then output to a file with full paths to each decoy or structure for further analysis, and if the user wishes, the top scoring poses may be copied to a directory. In addition, an energy vs. RMSD calculation for the full protein or each region can be performed using the PDBList compared to the loaded pose.

Finally, a help menu is available with links to the RosettaCommons bug tracker, the RosettaCommons user forum, general Rosetta manuals, as well as help for specific GUI tasks and setup instructions for both SCWRL and PyMOL integration.

## Discussion

The PyRosetta Toolkit was designed for ease of use and modification by incorporating the simplicity of PyRosetta and Python's Tkinter Application Programming Interface (API). We hope that the community will adopt and expand the GUI as a way to interface with their own applications, classes, and scripts. Long-term future projects may involve an interface of the PyRosetta Toolkit with the Chimera Suite, and/or the creation of a native C++ GUI. The lab will continue to develop the GUI for use with the growing number of applications and tools in Rosetta, while adding functions and tools to aid in molecular modeling and design as a whole.

## Methods

The GUI was written in python, using ActiveState's Komodo 6 Integrated Development Environment (IDE). The Netbeans (Oracle) and Eclipse (Eclipse Foundation) IDE's were used to explore, edit, and debug Rosetta C++ code where necessary. The open-source Clang LLVM compiler was used to compile PyRosetta binaries from the Rosetta C++ source code during development. Subversion (Apache Software Foundation) and Git (open-source) were used for version control. The GUI was tested on Ubuntu linux release versions 10.04 LTS and 12.04 LTS (Canonical, Ltd) as well as Apple Mac OS 10.6, 10.7, and 10.8.

The PyRosetta Toolkit and RosettaFlagFileBuilder require Python 2.6 or higher and are included in both the precompiled PyRosetta binaries available at www.pyrosetta.org (/GUIs directory) and Rosetta version 3.5. Complete documentation for the GUIs can be found at http://www.pyrosetta.org/documentation and within the documentation directory each GUI. A sub project in the Rosetta Mantis Bugtracker (bugs.rosettacommons.org) is available for toolkit-specific bugs and feature requests.
